# Supplementary material for: Highly Flame-Retardant and Low Heat/Smoke-Release Wood Materials: Fabrication and Properties
Source: Polymers (Basel). 2022 Sep 21;14(19):3944. doi: 10.3390/polym14193944 (PMC9571555; doi:10.3390/polym14193944)
Supplement: Supplementary file 1 [file polymers-14-03944-s001.zip › polymers-1813154-supplementary.pdf]

Supporting Information

Highly flame-retardant and low heat/smoke-release wood materials: Fabrication and properties

Ze-Peng Deng, Teng Fu*, Xin Song, Zi-Li Wang, De-Ming Guo, Yu-Zhong Wang and Fei Song*

The Collaborative Innovation Center for Eco-Friendly and Fire-Safety Polymeric Materials (MoE), National Engineering Laboratory of Eco-Friendly Polymeric Materials (Sichuan), State Key Laboratory of Polymer Ma-terials Engineering, College of Chemistry, Sichuan University, Chengdu 610064, China.

* Corresponding author. E-mail: Futeng@scu.edu.cn, songfei520@gmail.com

Table S1. Contrast information on flame retardant properties between this work and other fire retardant treated wood published in recent years.

Materails	retardant or coating	PHRR reduction (%)	THR reduction (%)	Ref.
Poplar	AHP-5/MUF	46	31	**29**
Wood	PTDP/SiR	23	/	**30**
Cunninghamia lanceolata	PEI/APP/Cu^{2+}	33	21	**31**
Birch	Mg–Al LDH	40	49	**17**
Chinese firs	SPS/boric acid	12	25	**32**
Plywood	MPEA	41	29	**9**
Wood	DGELU/DFA	56	12	**33**
Pinus sylvestris	CBP/X-1	63	12	**This work**
